# Acute Pancreatitis, Hypertriglyceridemia, and Diabetic Ketoacidosis: A Life-Threatening Triad

**DOI:** 10.7759/cureus.45631

**Published:** 2023-09-20

**Authors:** Adetola F Oshikoya, Nikita Kumari, Manita Bai, FNU Suman, Muhammad Haseeb

**Affiliations:** 1 Medicine, Near East University, Nicosia, CYP; 2 General Practice, General Hospital Odan, Lagos Island, Lagos, NGA; 3 Internal Medicine, Shaheed Mohtarma Benazir Bhutto Medical University, Larkana, PAK; 4 Internal Medicine, Allama Iqbal Medical College, Lahore, PAK; 5 Internal Medicine, Bahria International Hospital, Lahore, PAK

**Keywords:** diabetic ketoacidosis, acute pancreatitis, hypertriglyceridemia, type 2 diabetes mellitus, dka, metabolic complications

## Abstract

Hypertriglyceridemia (HTG)-induced pancreatitis is a known complication of uncontrolled diabetes mellitus (DM). However, the coexistence of diabetic ketoacidosis (DKA) and acute pancreatitis in the presence of HTG is rare and presents diagnostic and therapeutic challenges. We present the case of a 42-year-old female with poorly controlled type 2 DM who developed severe HTG-induced pancreatitis complicated by DKA. She initially presented with abdominal pain, metabolic acidosis, and marked hyperglycemia. Subsequent investigations revealed significantly elevated serum triglyceride and lipase levels and characteristic findings of acute pancreatitis on imaging. This case report highlights the complex interplay of metabolic disturbances in diabetes and the importance of timely recognition and tailored management to achieve a successful outcome.

## Introduction

Acute pancreatitis is a complex and often debilitating disease characterized by pancreas inflammation. The severity of acute pancreatitis can range from a mild, self-limiting disease to serious life-threatening conditions associated with systemic complications [1]. Acute pancreatitis has various etiologies, with gallstones and alcohol consumption accounting for the majority of the cases in developed countries [2]. However, the association between hypertriglyceridemia (HTG) and acute pancreatitis has gained increasing recognition in recent years. HTG-induced acute pancreatitis is a well-recognized entity primarily associated with uncontrolled diabetes mellitus (DM) [3]. However, the concurrent occurrence of diabetic ketoacidosis (DKA) in such cases is rare and presents unique challenges in terms of diagnosis and management. The convergence of HTG, acute pancreatitis, and DKA represents a complex clinical triad, necessitating comprehensive understanding, timely recognition, and tailored management [4,5]. In this report, we discuss a case of HTG-induced acute pancreatitis complicated by DKA and describe its clinical implications.

## Case presentation

A 42-year-old female with a longstanding history of poorly controlled type 2 DM presented to the emergency department with severe abdominal pain radiating to her back for the last two days. Associated symptoms included multiple episodes of projective vomiting and generalized weakness. The pain had started gradually and had been intermittent initially, followed by progressively worsening persistent pain. The patient also complained of frequent urination and excessive thirst but denied any history of dyspnea, dizziness, headache, or fever. She reported poor compliance with her DM medication for the last month due to financial constraints. Family history revealed that her mother also had DM in her 40s. She was not a smoker and denied any substance or alcohol abuse.

On examination, she was anxious, afebrile, irritable, hypotensive (95/70 mmHg), tachypneic (22/minute), and tachycardic (heart rate: 106/minute). Abdominal examination revealed tenderness in the epigastric area. Cardiovascular and respiratory system examinations were unremarkable. An electrocardiogram revealed sinus tachycardia and the chest X-ray was normal. Initial laboratory evaluations and arterial blood gas analysis are shown in Tables 1-2.

**Table 1:** Results of initial blood and biochemical parameter tests

Lab test	Result	Reference value
Serum lipase	2,900 IU/L	0–50
Total leukocyte count	10,000/microliter	4,000–11,000
Hemoglobin	11.1 g/dL	13.5–17.5
Creatinine	1.01 mg/dL	0.5–1.2
Random blood sugar level	497 mg/dl	<200
Urinary ketones	4+	Nil
Alanine aminotransferase	58 U/L	7–56
Aspartate aminotransferase	43 U/L	8–38
Alkaline phosphatase	87 U/L	45–115
Serum amylase	1,902 IU/L	<140
Serum sodium	140 meq/L	135–145
Serum potassium	4.3 meq/l	3.5–4.5
Chloride	106 meq/l	96–106
Serum ketone	3.4 mmol/l	<1.5
Anion gap	26	

**Table 2:** ABG analysis ABG: arterial blood gas analysis; Pa: partial pressure; HCO: bicarbonate

Lab test	Patient value	Reference range
PaO_2_	79 mmHg	75–100
pH	7.25	7.35–7.45
PaCO_2_	38 mmHg	35–45
HCO_3_	12 meq/l	22–24

**Figure 1:**
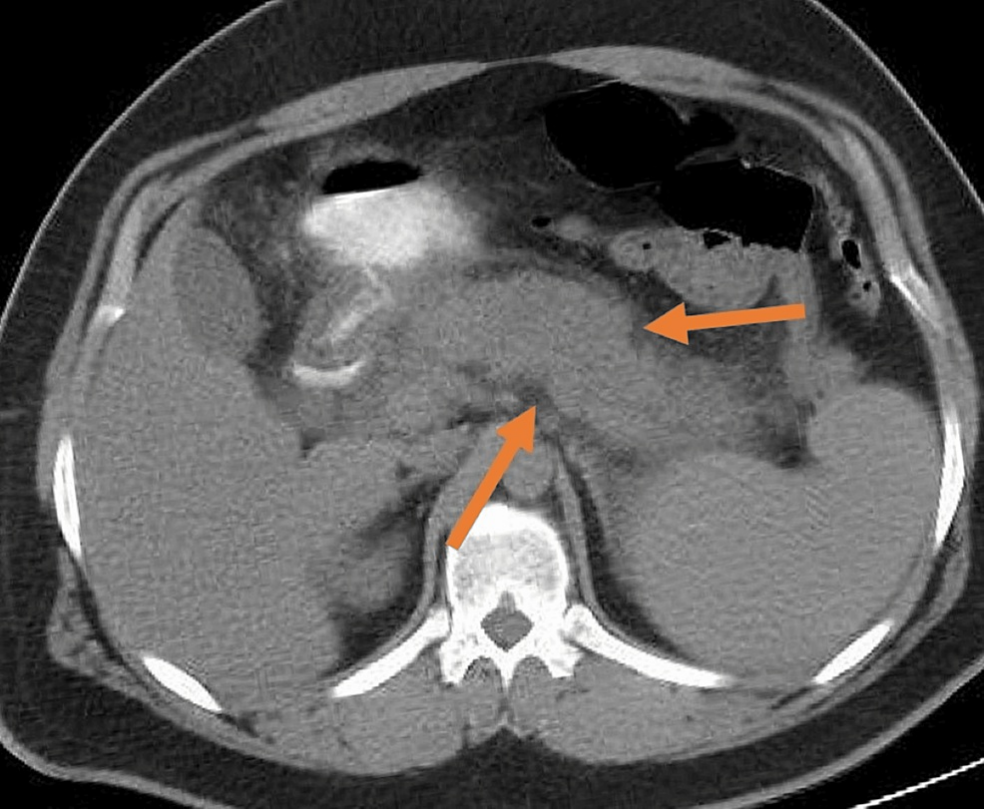
CT abdomen revealing diffuse inflammation of the pancreas with ill-defined borders and fat stranding CT: computed tomography

The presence of ketonuria and metabolic acidosis was suggestive of DKA. However, subsequent evaluations revealed HTG of 2670 mg/dl (normal range: <150 mg/dl). An abdominal CT revealed diffuse pancreatic enlargement with irregular borders (Figure 1). A provisional diagnosis of HTG-induced acute pancreatitis was made, further complicated by DKA.

**Figure 2:**
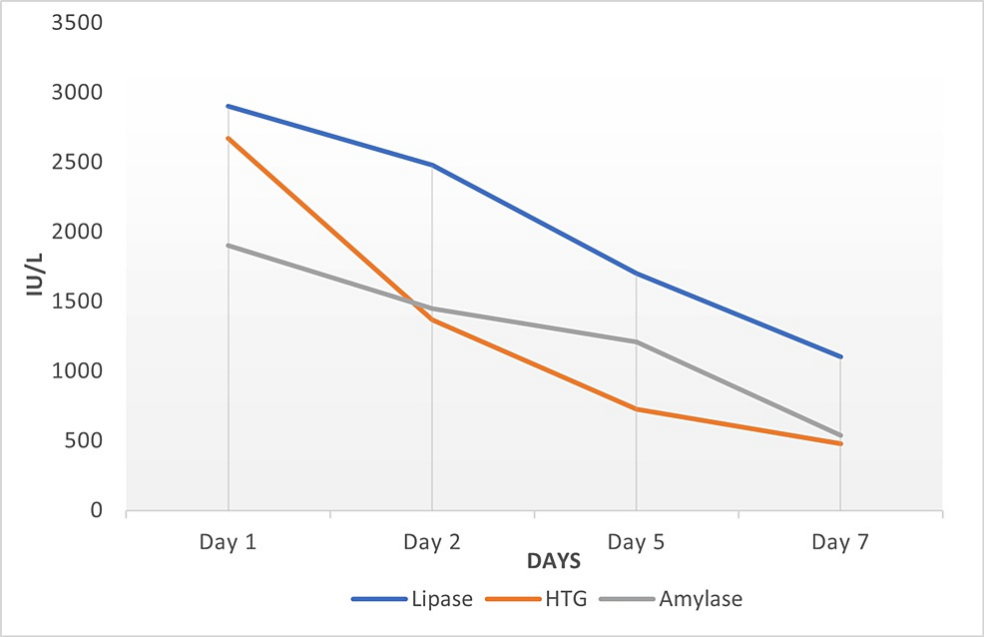
Serum HTG, lipase, and amylase levels during hospital stay HTG: hypertriglyceridemia

The patient was promptly admitted to the ICU and managed with intravenous hydration, analgesia, and insulin infusion. Aggressive fluid resuscitation, electrolyte correction, and nutritional support were instituted along with low molecular heparin. One session of plasmapheresis was performed to reduce the serum triglyceride level. Her clinical status improved gradually with the resolution of abdominal pain and vomiting. Serum triglyceride, serum lipase, and amylase levels normalized (Figure 2). She was transitioned to subcutaneous insulin therapy and received diabetes education to ensure improved adherence to medication upon discharge. The patient was discharged after two weeks of hospitalization with close follow-up arrangements.

## Discussion

Our case report highlights an uncommon presentation of HTG-induced acute pancreatitis with concurrent DKA in a female patient with uncontrolled type 2 DM. Although these conditions have been separately associated with acute pancreatitis, their simultaneous occurrence in a single patient is uncommon [6]. A recent study involving a cohort of 48 patients diagnosed with acute pancreatitis, DKA, and HTG revealed that DKA in acute pancreatitis is mainly triggered by high levels of HTG, which exacerbated HTG levels. Patients diagnosed with acute pancreatitis and DKA had higher levels of HTG and more extended hospital stays and complications compared to those without DKA [7]. Narala et al. have described a case of acute pancreatitis induced by HTG concurrent with DKA as an initial manifestation of type 2 DM [8]. Naqvi S et al. reported a case of the life-threatening triad of acute pancreatitis, DKA, and HTG due to poor compliance with DM treatment measures [9].

The pathophysiology of HTG-induced acute pancreatitis complicated by DKA involves a complex interplay of metabolic disturbances that can lead to severe pancreatic inflammation and systemic complications. The pathophysiology of HTG-induced acute pancreatitis is triggered when the serum triglyceride level exceeds 1000 mg/dl [10]. There are various pathways that can trigger acute pancreatitis in the presence of DKA. Pancreatic lipases are responsible for breaking down triglycerides into free fatty acids (FFAs) and glycerol. In hypertriglyceridemia, the elevated levels of triglycerides overwhelm the capacity of pancreatic lipases, leading to the hydrolysis of triglycerides into FFAs within the pancreas and the bloodstream, surpassing the binding capacity of plasma proteins. FFAs join to form micelles, which act as detergent-like properties leading to cellular injury, oxidative stress, and mitochondrial dysfunction [11]. These toxic FFAs initiate an inflammatory cascade within the pancreas. The release of proinflammatory cytokines and chemokines, such as interleukin-1β (IL-1β), tumor necrosis factor-alpha (TNF-α), and interleukin-6 (IL-6), contributes to the development of pancreatic inflammation [12]. DKA is another life-threatening condition arising from insulin resistance, which further exacerbates HTG. Eventually, HTG-induced acute pancreatitis complicated by DKA involves a cascade of events driven by elevated triglycerides, toxic FFAs, inflammatory responses, insulin deficiency, and metabolic disturbances. The interactions between these components make the clinical presentation complex and challenging to manage, requiring a multidisciplinary approach and tailored treatment strategies [4].

The management of HTG-induced acute pancreatitis primarily involves reducing serum triglyceride levels. This can be achieved through dietary modification, lipid-lowering medications, and, in severe cases, therapeutic plasma exchange or plasmapheresis [7,8]. Intravenous hydration, pain management, and nutritional support are essential components of pancreatitis management [9]. In HTG-induced acute pancreatitis, intravenous insulin can be used for the treatment of severe HTG and it is associated with simple initiation and generally good patient tolerance. The mechanism of action involves insulin's ability to reduce triglyceride levels by enhancing the activity of lipoprotein lipase (LPL). This enzyme, in turn, catalyzes the hydrolysis of TG into fatty acids and glycerol, facilitating the storage of these fatty acids within adipocytes. Additionally, insulin exerts an inhibitory effect on hormone-sensitive lipase in adipocytes, which is the pivotal enzyme responsible for the breakdown of adipocyte TG and the release of FFAs into the bloodstream [11]. The management of DKA is well-established and is centered on insulin therapy, fluid resuscitation, and electrolyte correction. However, in the context of HTG-induced pancreatitis, insulin resistance can be more severe, necessitating higher insulin doses and vigilant glucose monitoring [12,13]. The prognosis for patients with HTG-induced pancreatitis complicated by DKA is variable and dependent on multiple factors, including the severity of the pancreatitis, the degree of metabolic derangement, and the timeliness of intervention. Early recognition and prompt management are associated with improved outcomes [14].

## Conclusions

HTG-induced pancreatitis complicated by DKA represents a rare but clinically challenging triad of conditions. Clinicians should be vigilant about the potential coexistence of HTG-induced pancreatitis and DKA in patients with poorly controlled diabetes. Early recognition and tailored management are paramount in achieving optimal outcomes in these complex cases. Further research is needed to elucidate the underlying mechanisms and identify strategies for preventing and treating this life-threatening triad.
